# Evaluation of the Effects of Cement and Lime with Rice Husk Ash as an Additive on Strength Behavior of Coastal Soil

**DOI:** 10.3390/ma14051140

**Published:** 2021-02-28

**Authors:** Zahraalsadat Eliaslankaran, Nik Norsyahariati Nik Daud, Zainuddin Md. Yusoff, Vahid Rostami

**Affiliations:** Civil Engineering Department, Engineering Faculty, Universiti Putra Malaysia, 43300 Seri Kembangan, Malaysia; nina_5950@yahoo.com (Z.E.); zmy@upme.du.my (Z.M.Y.); vahid65r@gmail.com (V.R.)

**Keywords:** coastal soil, rice husk ash (RHA), physical properties, strength, pozzolan, stress

## Abstract

Coastal accretion and erosion are unavoidable processes as some coastal sediments undergo modification and stabilization. This study was conducted to investigate the geotechnical behavior of soil collected from Bagan Lalang coast and treated with lime, cement, and rice husk ash (RHA) to design a low-cost alternative mixture with environmentally friendly characteristics. Laboratory tests were carried out to analyze the physical properties of the soil (Atterberg limits and compaction properties), together with mechanical characteristics (direct shear and unconfined compressive strength (UCS) tests) to determine the effect of different ratios of stabilizer/pozzolan on the coastal soil and the optimum conditions for each mixture. Part of the purpose of this study was also to analyze the shear behavior of the coastal soil and monitor the maximum axial compressive stress that the treated specimens can bear under zero confining pressure. Compared to the natural soil, the soil treated with lime and rice husk ash (LRHA) in the ratio of 1:2 (8% lime content) showed a tremendous increase in shear stress under the normal stress of 200 kPa. The strength parameters such as the cohesion (c) and internal friction angle (ϕ) values showed a significant increase. Cohesion values increased considerably in samples cured for 90 days compared to specimens cured for 7 days with additional LRHA in the ratio of 1:2 (28%).

## 1. Introduction

In order to develop a country, it is necessary to improve its construction industry. As demand for construction material with low-cost and environmentally friendly nature rises, the innovation in designing new mixtures has grown. Contrarily, agricultural waste, in particular rice husk ash (RHA), has raised environmental concerns as the ash causes many health issues for local inhabitants. Another problem in today’s highly industrialized world is protecting the atmosphere from harmful contaminants associated with waste generation and disposal. Recently, various methods have been developed to turn hazardous waste into non-toxic compounds or to reduce the possible release into the atmosphere of toxic substances [1]. Silty sand soil is classified as a problematic soil in terms of its shear and compressibility. This type of soil covers most coastal areas and can easily be eroded and transferred to different locations. An investigation into the properties of the respective coastal sediments is needed. These quaternary, soft, fluvial, and shallow marine deposits can create geotechnical problems such as liquefaction, swelling, coastal erosion, and adversely affect the adopted constructions and industries in this area.

Soil improvement and stabilization techniques are mainly based on the type of added agents [2]. These are old techniques that have been in practice for a long time. The application of lime for soil stabilization began in 1945 in the United States [3]. Xunli Jiang [4] and Muntohar [5] had analyzed the strength development process on soil treated with lime and RHA, using three types of soil (soft soil, RHA–lime soil, and lime-stabilized soil). It has been discovered that RHA can effectively improve the shear resistance and water resistance of stabilized soil. Other scientists also conducted research about the improvement of various types of soil with lime [6,7]. This revealed that the compressive strength of unstable soil increases with the addition of lime, but only up to a certain quantity of lime, at which point it decreases as further increases in the amount of lime lead to a reduction in the reaction between the soil and the lime. In 2017, Heidemann [6] studied the stabilization of sedimentary silty-sand soil using cement and lime. The author noted that there was a decrease in strength with the curing time. Moreover, the presence of organic matter and the formation of sulfates can be responsible for the low strength and decay of soil.

In humid areas, lime itself cannot be considered to be the right stabilizer. In addition, the reaction between the lime and soil is prolonged. If the mixture is not balanced, it will fail to perform optimally [8,9]. Therefore, to reduce the cost and amount of lime used for stabilization purposes and expedite the chemical reaction between clay particles and lime, researchers started using specific alternative agents such as rice husk ash (RHA) or micro silica fume [10]. RHA is also composed of a high amount of silica, which can activate the soil/lime reaction [11]. Alhassan [11,12] studied the effects of lime and rice husk ash on clayey subgrades’ engineering properties. It was found that the addition of RHA as an agent affects the plasticity index and dry density, thereby improving the soil strength. Ye and Nguyen [13] studied the effects of changing the lime/RHA ratio and curing time. Nguyen [13] found out that RHA is of high pozzolanic activities because of the presence of amorphous silica, fineness, and high specific surface area. The effect of the curing period on the Atterberg limits and compaction properties for the improvement of coastal soil was also studied by other researchers such as Rahman [14]. The author indicated an increase in compressive strength by increasing cement content and curing period during unconfined compressive strength (UCS) tests. Rahman found that liquid limit and permeability were also altered as curing periods were extended from three to seven days.

This study’s objectives were to find an alternative for cement to strengthen coastal soil sediments and determine the effects of different lime ratios to rice husk ash (RHA) on coastal soil mixtures and, therefore, changes to the strength behavior of coastal soil. Considering that cement is not economically available, the use of RHA in cement products, even in developing countries, decreases the cost of materials and reduces the environmental risk associated with the existing open-field husk combustion in rice-producing countries. RHA’s broad prospect of application combined with other agents instead of cement can be expected due to the benefits of cost savings and environmental benefits associated with waste material disposal and reduced damage to the environment [15]. The work detailed within this technical paper is concerned with comparing the unconfined compressive strength (UCS) behavior of soil treated with various portions of lime and rice husk ash. Cement and lime are traditional curing agents. These agents have previously been used for stabilization purposes. Still, the high cost of cement and the negative impacts on the environment [16,17] have resulted in searching for a cost-effective and environmentally friendly solution as a replacement. Therefore, the lower cost of lime compared with cement, which is associated with the use of environmentally friendly material [18], has led to choosing lime and rice husk ash (LRHA) as pozzolanic waste for the stabilization and improvement of coastal soil properties. There are only a few studies focused on the coastal soil in terms of stabilization purposes. Since cement is considered the primary material currently used as a stabilizer in the construction and railroad industry, this research looked for a mixture of material that is similar but cheaper and greener. Lime is an economically viable option with a similar composition to cement, but its effect on soil stability is not comparable to cement. Hence, in this research, a mixture of industrial and agricultural waste has been added to the soil to create a replacement for cement.

## 2. Materials and Methods

**Figure 1:**
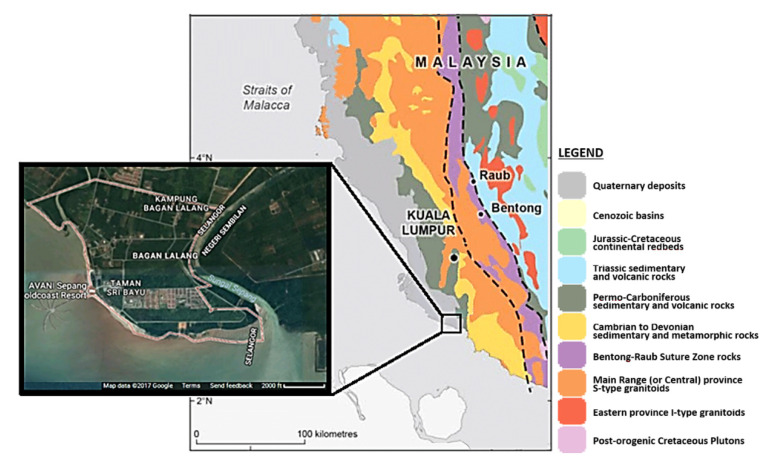
Bagan Lalang geological map, satellite image. The study area is bounded by the latitudes 2°, 28’-32.142”-2° 35.828’ N and longitudes 101°41.557’-101° 50’ 18.4374” E.

The coastal areas of Peninsular Malaysia are mainly composed of lowlands covered by soft alluvial and unconsolidated to semi-consolidated deposits consist of sand, silt, and clay. Most of the deposits are determined as Quaternary (Pleistocene Epoch) and occupy more than 20% of mainly coastal lowlands of both east and west coasts of the peninsula (Figure 1). Soil from Bagan Lalang on the west coast of Malaysia was used for the study. The coast is covered by tidal flats, mangroves, and deltas.

### 2.1. Soil Characteristics

**Figure 2:**
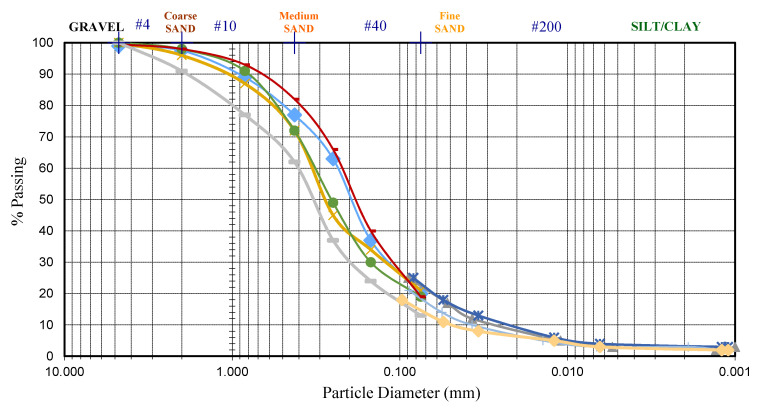
The particle size distribution of index soil sample collected from the Bagan Lalang beach site.

The particle size distribution curve of the coastal soil according to ASTM D422 [18] and ASTM D7928 standard method [19] is shown in Figure 2.

To measure the grain size distribution of soil samples taken from the west coast-Port Dickson, they were dried in a drying oven (this speeds up the drying process) and the samples were sorted into different grain sizes. To determine the soil moisture content, wet samples were weighed before and after the drying process to calculate the soil water content. In this test, a set of sieves was used, which grade from coarse to very fine, as well as a shaker table to do the labor. Samples were sieved for 5 min.

When the sieving was finished, the fraction from each sieve was collected separately. Each sieve’s contents were weighed and recorded. In order to find the percentage of very fine sand, silt, and clay (spheres particles finer than Sieve #200), the hydrometer method was used. This method is based on Stoke’s law, measuring the soil particles dropping speed of soil particles inside the water.

The grain size analysis shows that the sediment on the west coast of Malaysia consists of more than 90% fine soil, including 65–70% sand and 30–35% silt and clay particles. The coefficient of uniformity, Cu, was 6.538, which is considered well-graded sand, and the coefficient of curvature (Cc) was 3.040.

**Figure 3:**
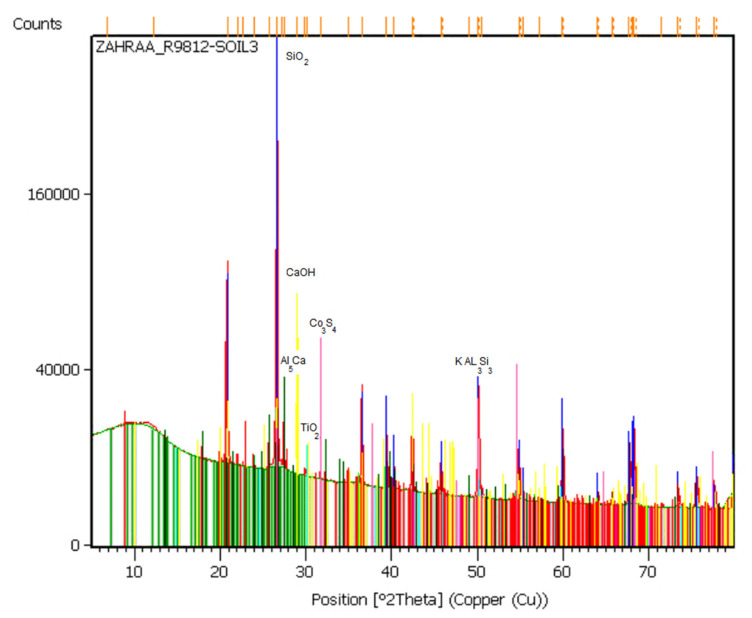
X-ray diffraction pattern of natural soil.

The principal mineral composition of the soil is marked on the X-ray diffraction pattern shown in Figure 3.

The physical properties of the coastal soil were also studied (Table 1). According to the unified soil classification system (USCS), the soil can mostly be classified as ML (low in silt) to OL (low in organic material, fine sand/silt plus organic silt and organic silty clays). Other parameters, including moisture content, soil consistency, and compaction, were obtained through laboratory tests explained in Section 2.3.

### 2.2. Stabilizer Materials

#### 2.2.1. Cement

Portland cement (PC) is the most commonly used cementing agent in the concrete building industry and was first made in 1825. The cement used in this project was a Type I ordinary Portland manufactured by YTL Sdn Bhd. (Kuala Lumpur, Malaysia), based on Malaysia Standards MS 522. The basic components of PC are calcium, silicon dioxide, aluminum oxide, and iron. It is a combination of limestone, gypsum, and shale or clay.

The primary and most important soil reactions with cement are the hydration reactions with the soil’s water content. This reaction results in forming the cemented material (calcium silicate and aluminate hydrates as in concrete).

Hydration of Portland cement [20] is as follows:

3CaO·Al_2_O_3_ + 6H_2_O → 3CaO·Al_2_O_3_·6H_2_O (1)

After the first day, the strength gain is due to the (3CaO SiO_2_) compound. The reaction that takes place is as shown in Equation (2):

2 (3CaO·SiO_2_) + 6H_2_O → 3CaO·2SiO_2_·3H_2_O + 3 Ca(OH)_2_ (2)

The final product of cement hydration form is Tri-calcium disilicate hydrate and is the principal binding material. When the 2CaO·SiO_2_ compound is hydrated, it contributes to the long-term strength given by a cement binder, the compound hydrated according to the following equation.

2(2CaO·SiO_2_) + 4H_2_O → 3CO·2SiO_2_·3H_2_O + Ca(OH)_2_ (3)

#### 2.2.2. Lime

Lime is commonly used as a stabilizer for chemical reactions. Lime is considered a cost-effective and environmentally friendly stabilizer to replace with Portland cement [16,18]. Lime is particularly beneficial for this study since it leads to cheaper construction, reduces environmental damage, and preserves the most highly qualified materials for priority uses. Hydrated lime for this study was purchased from MCB Sdn. Bhd. (Perak, Malaysia). During the pozzolanic process, the pozzolan paste (C–S–H and C–A–H) formed in chemical equilibrium, which leads to secondary reactions with any other reactive materials such as clays, RHA, or pozzolans within the soil. The chemical reaction in lime is as follows:

Ca(OH)_2_ + SiO_2_ → CaO·SiO_2_·H_2_O (C–S–H) (4)

Ca(OH)_2_ + Al_2_O_3_ → CaO·Al_2_O_3_·H_2_O (C–A–H) (5)

In fact, during the soil stabilization process, pozzolanic reactions occur between hydrated lime, silica, and alumina of the clay minerals to produce cementing material, including calcium silicate-hydrates calcium alumina hydrates [21].

Table 2 shows the physical and chemical composition of cement, lime, and RHA used in this study.

#### 2.2.3. Rice Husk Ash (RHA)

RHA is an industrial pozzolan and waste of rice production, which is abundant in countries like Malaysia. The RHA has been used in this research, supplied by Bernas Sdn. Bhd., Tanjung Karang Malaysia. RHA, made from rice husk (RH)-controlled burning, has an average silica content of about 90%. Rice husk has a fairly uniform chemical composition. The rice husk burning process is expected to eliminate only the organic contents and remain as silica in an amorphous shape. The balance is Al_2_O_3_, CaO, Fe_2_O_3_, MgO, and K_2_O. The amount of silica in rice husk depends on the variety of the rice, soil, and climate conditions, prevailing temperature, and agriculture practices.

The rice hush has low bulk density and high porosity. The ash’s chemical activity, particularly in combination with lime, is related to the form of silica in the ash and the carbon content.

Rice husk ash with high silica content reacts with binder elements to produce an optimum pozzolanic reaction. It happens by producing additional C–S–H (CaO·SiO_2_·H_2_O), called calcium silicate gel, in many of the voids around hydrated cement particles, such that silica can affect the compressive and flexural strength.

### 2.3. Laboratory Tests

The experimental research included mixing various percentages of lime and rice husk ash in the ratios of 1:1 and 1:2 to a mixture of oven-dried soil and water.

Before mixing the ingredients, lime and RHA material were sieved, passing through a sieve with a mesh size of 200 µm, to separate the coarse particles. At this stage, the soil was mixed with different percentages of lime:RHA (LRHA) and cement:RHA (CRHA) (4, 6, 8, and 10%) at and ratios of 1:1 and 1:2, and the mixing process continued until the soil and stabilizers showed a uniform color and texture. For each of the above mixtures, 4 samples were prepared. Specimens were cast in 50 mm × 100 mm standard cylindrical molds and were taken out after 1 h of casting. During the curing process, all the samples were kept in water till the specified date of testing. Compressive strengths of treated samples were determined at the ages of 7, 14, 28, and 90 days according to ASTM-D 1632 [22]. All the treated samples were prepared for UCS tests at their optimum moisture content (OMC).

#### 2.3.1. Atterberg Limits and Compaction Tests

The Atterberg limit test and compaction tests were conducted on both treated and untreated soil samples according to ASTM D4318 [23] and ASTM 1557 [24]. Each soil sample was prepared in a modified proctor 1 L mold. During the modified proctor test, soil mixtures were mixed with different moisture contents and allowed to be homogenized 24 h before compaction. Each layer of the soil sample was compacted by a hammer (4.53 kg weight), freely dropped from a height of 457 mm, blows were applied to the soil 25 times, and then the mold was filled again by the soil and the compaction procedure was repeated. During these tests, the plastic limit, liquid limit, and plasticity index for all the untreated soil and treated samples were determined.

The first series of compaction tests’ main goal was to determine the compaction characteristics of untreated soils. Secondly, the tests were conducted to determine the proctor’s compaction properties following stabilization in various lime, RHA, and cement proportions.

#### 2.3.2. Unconfined Compressive Strength

UCS test is one of the typical soil mechanical tests for soil stabilization applications, and it is known as a method for evaluating improvements to treated soil. UCS test on the treated samples was performed using ELE International, Unconfined Compression Tester KA-25–3602, according to ASTM D2166 standard method [25].

The experimental work was focused on investigating the effect of different percentages of lime and RHA (4,6, 8 and 10%, in portions of 1:2 and 2:2) on the shear strength of the soil and comparing it in various amounts of LRHA to find a suitable replacement for cement.

#### 2.3.3. Direct Shear Test

The direct shear test was carried out to determine the variation of shear strength, cohesion, and friction angle and the effect of different amounts of LRHA and CRHA on each parameter. The shear strength of the treated samples and untreated soil was tested using ELE direct shear apparatus and according to ASTM 3080 standard [26]. The samples were mixed with lime, RHA, and cement, poked, and compacted with the optimum amount of moisture until they reached the γdmax using the modified proctor test. To obtain a higher cohesion in the treated samples, different LRHA or cement amounts were mixed with the soil and treated for 7, 14, 28, and 90 days.

## 3. Results and Discussion

### 3.1. Effect of LRHA and CRHA on Plasticity and Compaction Parameters

The plasticity characteristics of soil are due mainly to the clay particles’ plate structure and lubricate bonding effect of adsorbing water around the clay particles [27].

**Figure 4:**
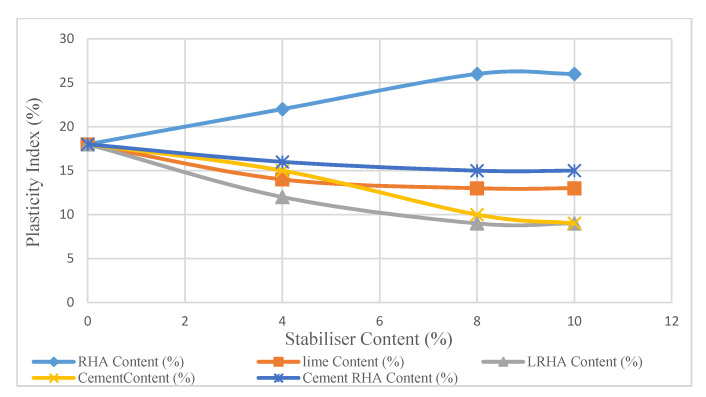
Effect of stabilizer contents on treated soil plasticity index after 14 days.

Figure 4 shows the modifying effects of stabilizers on the plasticity index of the samples treated with RHA, cement, lime, LRHA, or CRHA, where the soil plasticity index decreased by 45% in LRHA-treated soil. The plastic index of the untreated soil was 18%. The immediate effect of adding 4% of stabilizers was to reduce this value considerably. The plasticity index decrease was relatively faster in the soil/LRHA than in the soil/CRHA mixture. Reduction in plasticity indicates an improvement (Figure 4).

The addition of 6% and 8% stabilizer content decreased the plasticity index to values below those obtained with a 4% stabilizer. The plasticity index gradually reduced to 15% in soil treated by 8% of CRHA and then remained unchanged. In contrast, the plasticity index rate continued to drop to 9% for samples mixed with 8% of LRHA, and then it remained intact. In conclusion, it is obvious that the rate of plasticity index in the LRHA sample dropped throughout the whole tests and was at a lower level than the CRHA Figures.

**Figure 5:**
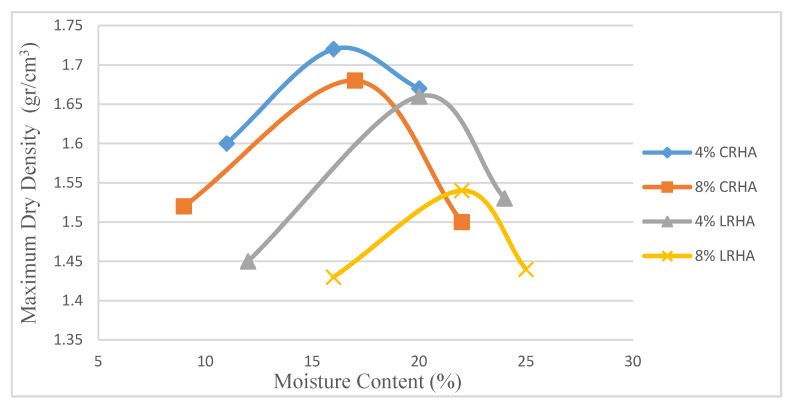
Maximum dry density (γdmax) based on stabilizer contents (1:2).

Figure 5 shows the maximum dry density (MDD) (γ dmax) and optimum moisture content (OMC) values based on the LRHA and CRHA content using a modified proctor test. Xunli Jiang and Phanikumar observed a similar trend while using RHA mixed with other agents. The reduction rate differs depending on the clay content and the stabilizer type [4,27].

As shown in Figure 5, the immediate effect of adding CRHA or LRHA stabilizers is to increase the maximum dry density values sharply. However, after moisture contents reach the optimum level, the MDD approaches the original values. Based on the compaction test (modified proctor), the MDD was inversely proportional to the OMC due to the size factor and the effect of circulation. In all samples, the maximum dry density of soil decreased with the addition of RHA content because of the lower specific gravity of RHA. On the other hand, the moisture content in RHA-treated soil continuously increased due to the pozzolanic reaction between RHA and soil, which needs more water. Treated soil with 4% CRHA showed the optimum moisture content of 16%, while when the CRHA content was increased to 8%, the OMC value rose to 17%. A similar pattern was shown in the LRHA mixture. In fact, when the stabilizer content was increased from 4% to 8%, the moisture content and MDD increased dramatically to a peak, demonstrating the optimum moisture content [4].

The samples treated with 8% LRHA showed a 7.8% decrease in MDD (γ dmax) values than the treated sample with 4% of LRHA. Similarly, the MDD showed a reduction of approximately 2.3% when the CRHA content increased from 4% to 8%. This reduction in MDD values could have been due to the pozzolanic reaction and water content.

### 3.2. Compression Strength Behavior

**Figure 6:**
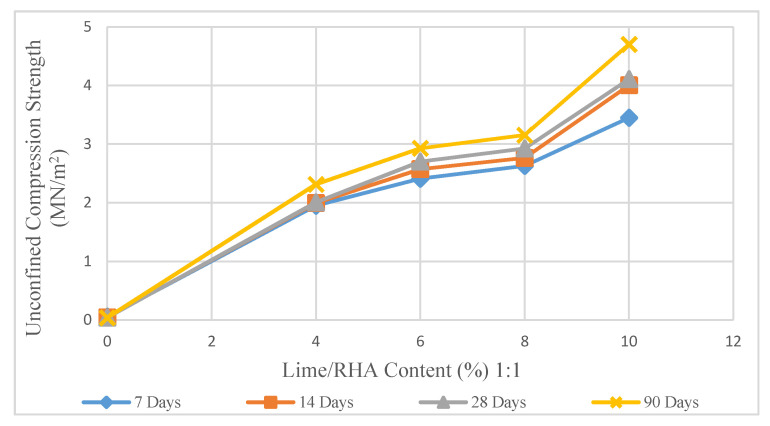
Effect of lime and rice husk ash (LRHA) content (1:1) on unconfined compression strength.

**Figure 7:**
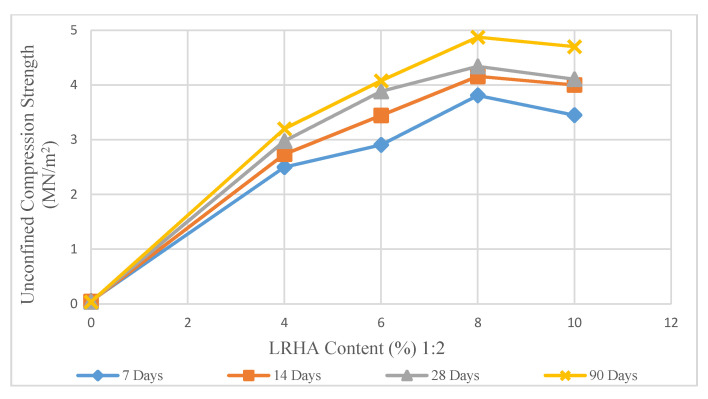
Effect of LRHA content (1:2) on unconfined compression strength.

**Figure 8:**
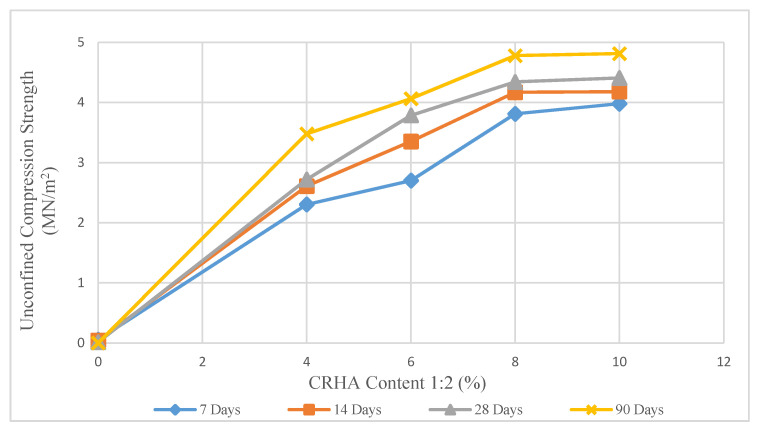
Effect of CRHA (1:2) content on unconfined compression strength.

The effect of the stabilizer content and curing duration on the UCS test is shown in Figure 6, Figure 7 and Figure 8. These figures show the changes in the UCS values for different stabilizer mixes in various curing periods of 7 to 90 days. A significant increase in compression strength values recorded in samples treated with LRHA.

Figure 6 shows apparent changes in the UCS values of soil/LRHA mixture (1:1) for curing time of 7 days to 90 days. As for all graphs, the UCS grew consistently from 0.038 to below 3.154 MN/m^2^ in 8% of LRHA content (1:1). The tendency increased and hit a peak of 4.702 MN/m^2^ in 10% of LRHA content.

Figure 7 shows that the UCS increased sharply, while the LRHA content increased to 8%. The maximum compression strength recorded was 4.875 MN/m^2^ in LRHA-treated soil at a ratio of 1:2 after 90 days of curing. The effect of additional LRHA on the UCS test showed a constant decrease in UCS values after stabilizer content exceeded 8%. In addition, the curing time of 28 and 90 days is shown to have a sharp influence on the treated soil’s strength behavior [1,15]. The maximum compression strength obtained from the LRHA treated samples is relatively close to what was found in similar research recently done by Liu [28] with a similar pattern and uprising trend.

In contrast, according to Figure 8, the samples treated with CRHA showed a continuous increase in UCS values, while the cement content was increased to 10%, with the UCS value rising gradually to 4.81 MN/m^2^ (cured for 90 days). The second-highest strength values were recorded from 8% CRHA samples at the ratio of 1:2, which was 4.4 MN/m^2^ after 28 days of curing (Figure 7). As has been expected, in each of the UCS tests, samples cured for 90 days showed higher compression strength values than samples cured for a shorter duration.

### 3.3. Shear Stress Behavior

**Figure 9:**
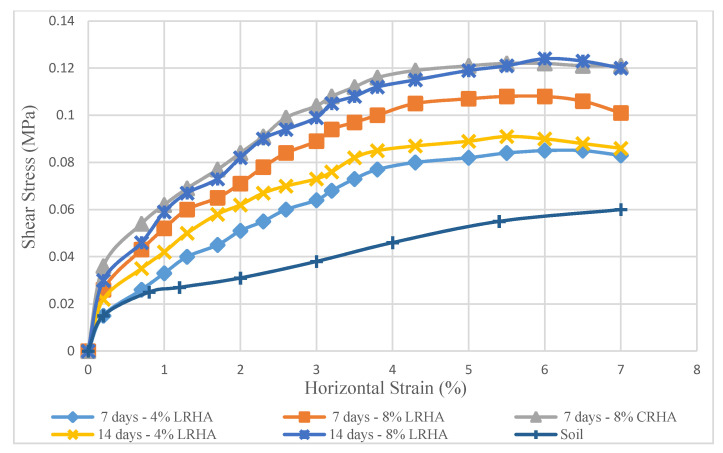
Shear stress to horizontal strains diagram—LRHA 1:2 (vertical stress 0.5 MPa).

Figure 9 shows the changes in shear stress (τ) versus horizontal strain on pure soil and specimens treated with 4 and 8% LRHA and CRHA at the ratio of 1:2, under normal stress of 0.5 MPa for curing periods of 7 days and 14 days.

The strain data accumulated from six tests, obtained from untreated soil and samples treated with LRHA in the lower level (4%) after 7 days curing, showed lower shear stress values than those treated with higher levels of LRHA and longer curing duration. A similar pattern was obtained by Phanikumar [27] but in higher levels of shear strength. The stabilizer material used was lime sludge mixed with cement.

According to Figure 9, LRHA treatment showed a significant increase in strength. This immediate improvement in strength led to better workability. By increasing the LRHA content from 4% to 8%, together with the curing time, the shear strength increased sharply, while the horizontal strain also increased. Meanwhile, when the LRHA content exceeded 8%, the samples suddenly became fragile at a strain of 6%. However, stress changes show that with increasing LRHA content from 4 to 8%, the shear stress increased dramatically.

### 3.4. Effect of LRHA and CRHA on Soil Cohesion and Internal Friction

**Figure 10:**
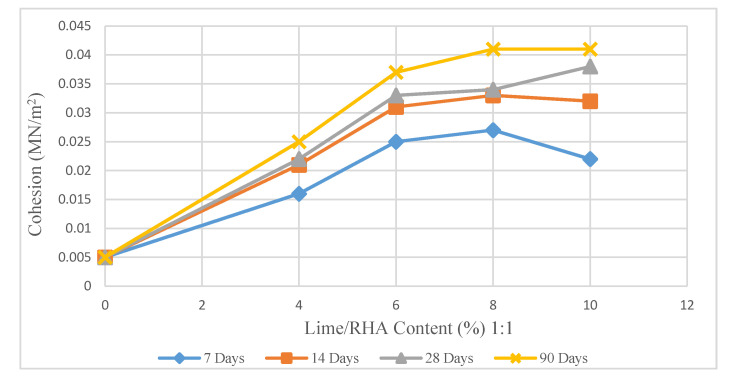
Effect of lime/RHA 1:1 content on cohesion.

**Figure 11:**
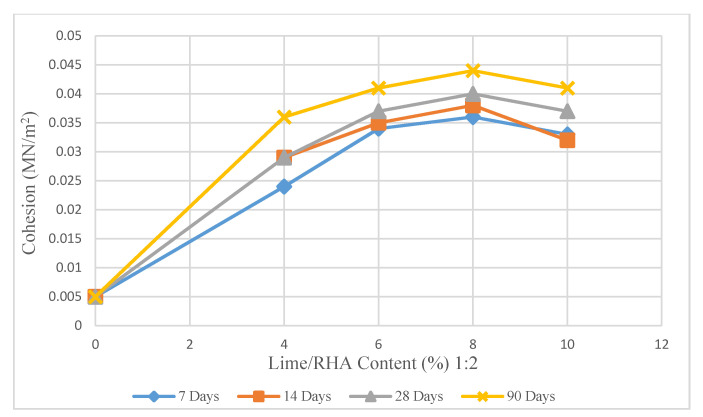
Effect of lime/RHA 1:2 content on cohesion.

**Figure 12:**
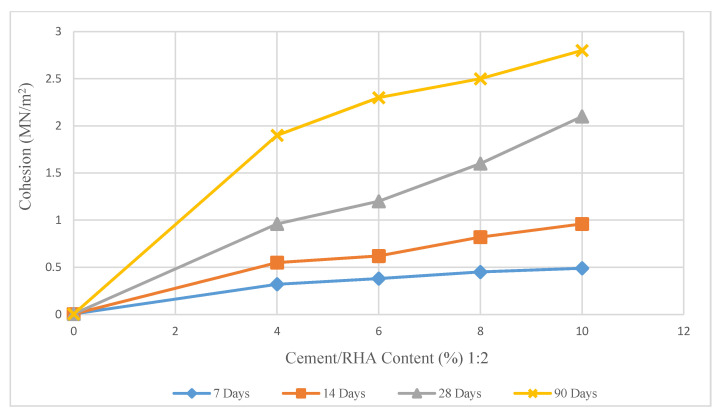
Effect of CRHA (1:2) on soil cohesion.

Figure 10, Figure 11 and Figure 12 demonstrate the changes in the cohesion of treated soil based on various agents. According to Figure 10, the soil sample treated with 8% LRHA in the ratio of 1:1 for 7 days of curing shows a significant increase in cohesion values (440%) compared to untreated samples and a sharp increase in cohesion of 33% compared to the samples treated with (8%) LRHA at a ratio of 1:2 after 7 days curing (Figure 11).

When the curing time increased to 90 days, the cohesion dramatically increased for all types of mixtures. When the lime content increased by more than 6%, the cohesion rose gradually with further addition of lime. A similar pattern was shown in research by Qiang [29]. The cohesion values obtained were lower due to using only lime as a stabilizer. The maximum cohesion recorded by the author was obtained using 6% lime and a loading rate of 8%/min. Similar to the findings of this research, Qiang showed that the cohesion values increased almost linearly with the increase in lime content. After cohesion reached its peak, with additional lime content, the values started dropping drastically.

As a result of mixing 4%, 8%, and 10% LRHA with soil in a ratio of 1:2 for 7, 14, 28, and 90 days of curing (Figure 10), there was an increase in cohesion values compared to the LRHA-soil mixtures in a ratio of 1:1 (Figure 9). As the curing time was extended to 90 days, the maximum cohesion value increased by 720% and 780% for 8% LRHA with soil in 1:1 and 1:2, respectively.

The important point regarding the samples stabilized with LRHA (1:2) is that the cohesion slightly decreased with the addition of more than 8% lime (Figure 10 and Figure 11). At the same time, there was an upward trend in the cohesion with the addition of LRHA (1:1). As seen, the cohesion of the soil sample treated with 8% lime/RHA at a ratio of 1:2 after 7 days of curing was approximately 620% more than that of the untreated soil (Table 2).

As shown in Figure 10 and Figure 11, the increase in cohesion was related to increased lime or LRHA content. This increase could have been due to the strong bonding between the binder elements and the aggregates. However, there was a dramatic increase in the cohesion at the later stages and in the samples cured and stabilized for a more extended period (from 7 days to 90 days) with the combination of lime and rice husk ash (ratio of 1:2). Furthermore, the combination of 8% lime + 16% RHA exhibited a high increase in cohesion beyond the curing period of 7, 14, 28, and 90 days. When the lime content exceeded 8%, the cohesion became steady and, in some cases, showed a slow increase.

The direct shear box test result of the samples treated with CRHA shows a tremendous increase in cohesion due to the stabilizer material. The cohesion values show a consistent trend with an increase in CRHA content. For 7 days and 14 days of curing, the cohesion increased gradually with further increases in the cement. The same change occurred in the lime-treated samples. However, after 28 days, the cohesion improved rapidly from 0.9 MN/m^2^ to 2.15 MN/m^2^ (cement content increased from 4% to 10%). A significant change occurred when the curing duration was extended to 90 days. The cohesion increased by 46% with the addition of 6% cement content (Figure 12).

**Figure 13:**
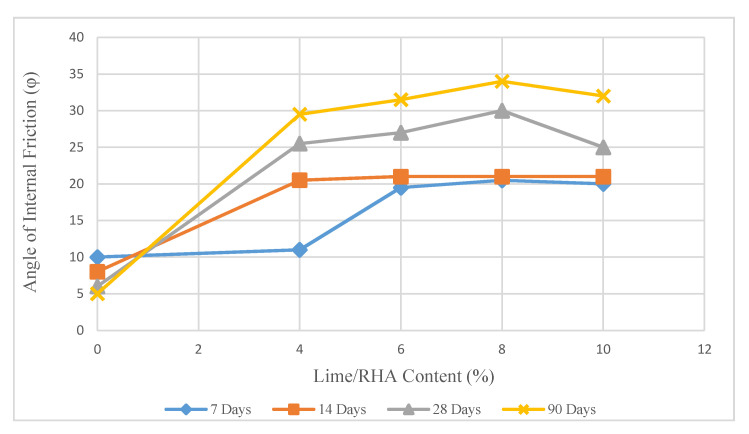
Internal friction angle in treated soil with LRHA 1:1.

**Figure 14:**
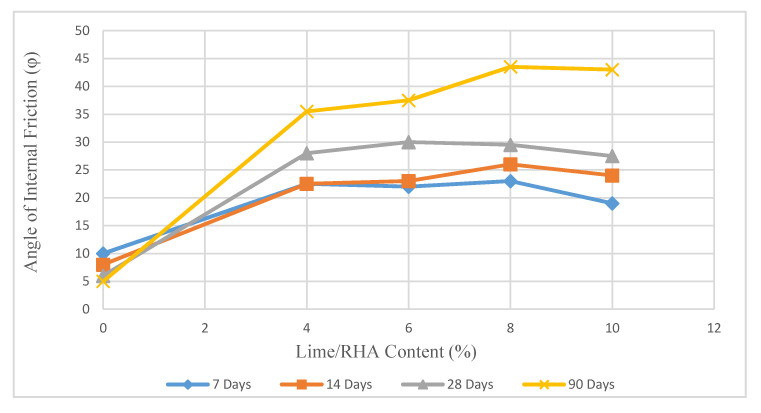
Internal friction angle in treated soil with LRHA 1:2

As shown in Figure 13 and Figure 14, the internal friction angle in samples stabilized with 8% lime/RHA in the ratio of 1:1 and 1:2 for 90 days, increased by 580% and 770%, respectively, compared to the untreated soil.

The increase in the internal friction angle can be explained by the flocculation of the soil particles with the addition of lime, following which the particle size distribution of the soil to a finer material and the pozzolanic reaction resulted in higher ϕ values.

Increasing the lime content in the LRHA mixture (1:2) to 8% led to a rise in the internal friction to 44° after 90 days of curing, which is the maximum internal friction angle recorded during all the tests. With a further increase in the lime content (more than 8%), the value of ϕ was relatively constant. When the curing time for the sample treated with 8% LRHA (1:1) increased from 7 days to 28 days, the angle of internal friction increased by about 10° (46%).

**Figure 15:**
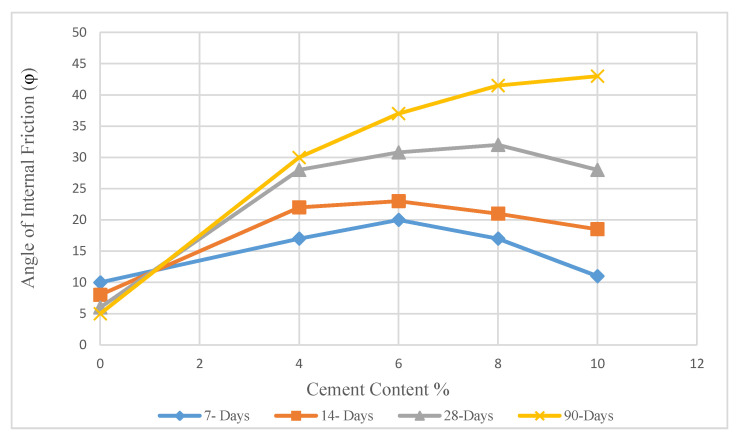
Internal friction angle in treated soil with CRHA 1:2.

Although the friction angle increased sharply in LRHA soil samples, the highest increase was recorded from soil samples treated with 8% CRHA after 28 days of curing compared to a similar specimen cured for 7 days (Figure 15). Qiang [29] showed the change curve of the cohesion and the internal friction angle under the curing period of 21 days. It demonstrated that the ϕ values reached the maximum when the lime content was 6% and gradually decreased. The changes in friction angle reported by Qiang are in accordance with the current findings.

## 4. Conclusions

The behavior of soil samples treated with lime/RHA and cement/RHA was investigated in this research. The detailed conclusion is as follows:

1. With a curing time extended to 28 days and 90 days and a higher level of LRHA up to 8% (1:2) mixture, there was a significant improvement in the optimum moisture content values (OMC) of about 10%.
2. The results derived from the UCS and direct shear tests suggest that increasing the LRHA content (8% lime and 16% RHA (ratio of 1:2 after seven days of curing) enhanced the maximum shear strength (τmax) up to 46%. The adhesion and internal friction (8% lime/RHA and 28 days of curing time) reduced up to 45% of the vertical soil displacement.
3. There was a dramatic increase in the internal friction (φ) to 38.5° (770% increase) and an increase of 58% in the shear stress (τ) of the samples treated with 8% Lime/RHA in the mixing ratio of 1:2 compared to the untreated soil. The maximum increase in φ and cohesion values were 44° and 0.44 MN/m^2^, which were obtained from samples treated with 8% LRHA in 1:2 ratio after 90 days of curing.
4. Cohesion values increased considerably by additional LRHA in the ratio of 1:2 for 7 days (33%) and 90 days (7%) compared to the samples treated with LRHA 1:1. In addition, there was an increase in cohesion values of 46% compared to the samples treated with (10%) LRHA in the ratio of 1:1 after 7 days of curing. The optimum cohesion and φ values are quite comparable and similar to the sample treated with cement/RHA.
5. The soil treated with LRHA in the ratio of 1:2 (8% lime content) showed a tremendous increase in shear stress (936.63%) at normal stress of 200 kPa, compared to the untreated soil after 90 days.
6. Overall, the soil mixture with 8% LRHA in the portion of 1:2 showed competitive and quite similar strength properties, including shear strength and cohesion, compared to samples treated with cement mixture. Hence, it is strongly recommended to replace lime with cement in coastal soil treatment.

## 5. Future Scope

- Only a strength study and simulation have been used to evaluate the coastline and coastal sediments. It is recommended to examine and simulate the model’s capability for the Bagan Lalang roadside area and the peat zone.
- It is recommended to study the effect of monsoon and rainfall on sediment texture and soil properties on the West Coast to study the sediments’ behavior during and after treatment.
- For more sensitive results, 2D/3D Analysis and simulation is recommended, considering the third-dimensional confining effect.

## Figures and Tables

**Table 1:** Physical properties of West Coast Malaysia coastal soil.

Properties	Unit	Value
**Grain size analysis**		
- Sand	%	65–70
- Silt and clay	%	30–35
USCS classification	-	ML/OL
Specific gravity	-	2.63
Moisture content	%	16.3
**Consistency limit**		
- Liquid limit	%	47.5
- Plastic limit	%	28
- Plasticity index	%	19.5
**Compaction study**		
MDD	mg/m^3^	1.48
OMC	%	16.2

**Table 2:** Cement, lime, and RHA chemical composition.

Chemical Composition	Unit	Cement	Lime	RHA
CaO	%	64.82	76.24	1.3
SiO_2_	%	6.72	0.21	93.67
Fe_2_O_3_	%	17.58	19.32	0.47
K_2_O	%	0.82	1.03	1.82
MgO	%	5.37	2.92	0.57
Al_2_O_3_	%	3.27	0.25	1.45
**Physical Properties**				
Grain specific gravity	Mg/m^3^	3.15	2.37	2.11
pH Value		13	12.4	7.10

## Data Availability

This paper is a part of an extensive Doctoral research project and all finding, materials and test methods is mentioned on the thesis. The PhD thesis will be sumbited to Universiti Putra Malaysia’s Library before end of 2021.
